# Association of Neighborhood Income with Clinical Outcomes Among Pregnant Patients with Cardiac Disease

**DOI:** 10.1007/s43032-022-00978-z

**Published:** 2022-07-11

**Authors:** Corinne Carland, Danielle M. Panelli, Stephanie A. Leonard, Eryn Bryant, Elizabeth B. Sherwin, Christine J. Lee, Eleanor Levin, Shirin Jimenez, Jennifer A. Tremmel, Sandra Tsai, Paul A. Heidenreich, Katherine Bianco, Abha Khandelwal

**Affiliations:** 1 Department of Medicine, University of Pennsylvania, Philadelphia, PA USA; 2 Division of Maternal-Fetal Medicine and Obstetrics, Department of Obstetrics and Gynecology, Stanford University School of Medicine, Stanford, CA USA; 3 Division of Cardiovascular Medicine, Department of Medicine, Stanford University School of Medicine, 300 Pasteur Dr. Rm A260, MC 5319, Stanford, CA 94305 USA

**Keywords:** Cardio-obstetrics, Women’s health, Women’s heart health, Social determinants, Pregnancy

## Abstract

Cardiovascular disease is the leading cause of pregnancy mortality. Socioeconomic and racial disparities in pregnancy are well established. Despite this, little is known about the impact of social determinants of health in pregnant patients with heart disease. This study aims to determine whether pregnant patients with heart disease living in lower income neighborhoods and managed at cardio-obstetrics programs have higher rates of cardiac events or preterm deliveries compared with those living in higher income neighborhoods. This is a retrospective cohort study of 206 patients between 2010 and 2020 at a quaternary care hospital in Northern California. The exposure was household income level based on neighborhood defined by the US Census data. Patients in lower income neighborhoods (*N* = 103) were 45% Hispanic, 34% White, and 14% Asian versus upper income neighborhoods (*N* = 103), which were 48% White, 31% Asian, and 12% Hispanic (*p* < 0.001). There was no significant difference in the rates of intrapartum cardiac events (10% vs. 4%; *p* = 0.16), postpartum cardiac events (14% vs. 17%; *p* = 0.7), and preterm delivery (24% vs. 17%; *p* = 0.23). The rates of antepartum hospitalization were higher for lower income neighborhoods (42% vs 22%; *p* = 0.004). While there is no significant difference in cardiac events and preterm delivery rates between patients from low versus high income neighborhoods, patients from lower income neighborhoods have higher antepartum hospitalization rates. Earlier identification of clinical deterioration provided by a cardio-obstetrics team may contribute to increased hospitalizations, which might mitigate socioeconomic disparities in outcomes for these pregnant patients with heart disease.

## Introduction

Cardiovascular disease is the leading cause of maternal mortality in the USA [1–3]. The physiologic changes that occur during pregnancy involve hormonal and vascular adaptations, which have a direct effect on cardiovascular function including on blood volume, cardiac output, heart rate, vascular resistance, and coagulation [4]. Such changes make pregnancy a particularly vulnerable time for individuals with existing heart disease [5].

This is of increasing importance because the incidence of pregnancies complicated by heart disease is rising. This increase is due to several factors including advancing maternal age, an increasing number of congenital heart disease children surviving to childbearing age, and a growing incidence of cardiometabolic risk factors in younger people including obesity, diabetes mellitus, and hypertension [6–8]. Additionally, pregnancy complications like preeclampsia and gestational diabetes are associated with increased risk for cardiovascular disease later in life [9–11]. For this reason, early identification and proper treatment of this patient population may have both immediate and downstream health effects [4]. Several professional associations have reinforced the need for these patients to be cared for by a multidisciplinary team dedicated to cardio-obstetrics [12–15]. Despite this, there is limited evidence examining risks, best practices, interventions, and outcomes in this population [16–20].

There is growing evidence of racial and socioeconomic disparities in pregnancy outcomes [3, 21]. These disparities are reflective of social determinants of health, where neighborhood and community factors such as access to education, economic opportunities, and transportation play an influential role in health outcomes [22]. Higher poverty rates are associated with higher maternal mortality, with people living in counties with high poverty rates having double the rate of maternal mortality compared with those in low poverty counties [23]. One study found that Black women have higher rates of antepartum hospitalizations [24]. There is evidence for racial disparities in cardiovascular-related complications in pregnancy, persisting even when corrected for socioeconomic status, healthcare access, and medical comorbidities [25, 26]. However, there is a lack of studies examining the impact of neighborhood factors on outcomes specifically in pregnant people with pre-existing heart disease, especially those who are cared for at a dedicated cardio-obstetrics program.

We sought to examine socioeconomic disparities in cardiac and obstetric outcomes among individuals with cardiac disease in pregnancy by comparing lower income versus higher income neighborhoods of residence. We hypothesized that cardio-obstetric patients who live in lower income neighborhoods experience higher rates of obstetric and cardiac complications compared with those who live in higher income neighborhoods. Further, we suspected that participation in our cardio-obstetrics program may attenuate some of this risk.

## Materials and Methods

### Study Population and Data Collection

We conducted a retrospective cohort study consisting of a population that included pregnant individuals with cardiac disease managed at our institution’s multidisciplinary cardio-obstetrics program who were seen between November 2010 and April 2020. All pregnant people with heart disease seen at this institution were referred to this program. These individuals had been evaluated as part of a multidisciplinary conference with high-risk obstetrics, anesthesia, cardiology, cardiovascular surgery, and nursing. Data were collected under approval by the Stanford Institutional Review Board and stored on a secure, web-based software platform [27, 28]. All index pregnancies, as defined as first-time deliveries at our health care center, were included for analysis.

**Fig. 1:**
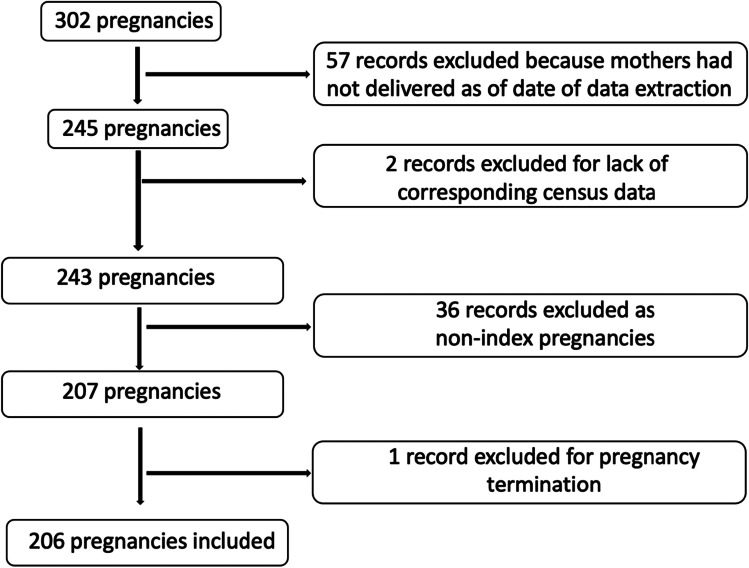
Flowchart of patients included and excluded for analysis. There were 302 pregnancies initially considered for inclusion. Pregnancies were excluded for not having been delivered yet, lacking census data, non-index pregnancies, and termination

There were a total of 302 pregnancy records. We excluded 57 records because delivery had not occurred as of the date of data extraction. Two records were excluded because the subjects’ recorded zip codes did not have a corresponding entry in the American Community Survey. Non-index pregnancies were excluded. There was a total of 207 (85%) index pregnancies and 36 (15%) subsequent pregnancies. There was one termination of pregnancy, which was excluded. In total, 206 pregnancies were included (Fig. 1).

Health and demographics information was extracted from electronic medical records using a standardized data collection form. Age was defined as age of mother at time of delivery. Race and ethnic categories were self-reported. Cardiac diagnoses were divided into five primary categories: structural (including aortic and valvular disease), cardiomyopathy, congenital, arrythmia, and other. Public insurance refers to Medicaid programs. Limited prenatal care was defined as less than 3 obstetric clinic prenatal visits. Late-to-care was defined as prenatal care initiated after 24 weeks’ gestation.

### Exposures and Outcomes

The exposure of interest was the median annual household income in the neighborhood of residence. Neighborhoods were defined using patient-reported 5-digit zip codes. The median annual household income is a value that the US Census Bureau reports. It is assigned using self-reported data obtained from the 2018 American Community Survey [29, 30]. Five-digit zip code tabulation area from the census data was matched and merged with recorded zip codes for each individual. Thus, a neighborhood median household income was assigned to each person. An overall median household income was defined based on the median value of incomes of included zip codes. Pregnancy records were divided into two categories (upper and lower) based on the overall median household income of the sample set. Individuals living in neighborhoods where the income level was above the median value were categorized as upper income neighborhoods, and those living in neighborhoods where the household income was below the median were categorized as lower income neighborhoods.

Primary outcomes were chosen to be consistent with the literature describing adverse outcomes in patients with heart disease in pregnancy [15, 31, 32]. The primary outcomes were (1) intrapartum or postpartum cardiac events and (2) preterm delivery. Cardiac events included pulmonary edema, chest pain, hypotension, arrythmia, heart failure, thrombotic events, hypoxia (oxygen saturation < 94%), myocardial infarction, spontaneous coronary artery dissection, aortic dissection, and cerebral vascular accident. Preterm delivery was defined as delivery before 37 weeks gestation and included both spontaneous and medically indicated preterm births. Secondary outcomes included preeclampsia, gestational diabetes, preterm labor, postpartum breastfeeding, postpartum hemorrhage, type of delivery, antepartum hospitalization, prolonged hospitalization postpartum (greater than 2 days for normal spontaneous vaginal delivery and greater than 4 days for cesarean delivery), neonatal intensive care unit (NICU) admission after delivery, small for gestational age (defined as weight below the 10th percentile for gestational age of a population-based reference [33]), and in-hospital maternal and neonatal death.

### Statistical Analysis

We calculated means with standard deviations for continuous variables and compared them between the upper and lower income groups using the Student’s *t* test. We calculated frequencies and percentages for categorical variables and compared them between the study groups using the Fisher’s exact test. *P* values < 0.05 were considered statistically significant. All statistical analyses were performed with R statistical software version 3.6.1 [34].

## Results

A total of 206 pregnancies were included (Fig. 1) from 109 different zip codes. The median household income per neighborhood of all the included records was $117,264 per household per year with a range of $40,452 to $250,000 + . The pregnancy records were stratified by neighborhood median income into upper income (*N* = 103) and lower income (*N* = 103) groups, which were compared by demographics and outcomes.

Table 1 describes the demographics and characteristics of the patients stratified by neighborhood income category. Individuals living in lower income neighborhoods had a lower mean age at delivery than those in the upper income neighborhoods (30.5 + / − 6.3 years vs. 33.7 + / − 5.1 years, *p* < 0.001). Individuals living in upper income neighborhoods were more likely to be White (*N* = 48%) and Asian (*N* = 31%), while those living in lower income neighborhoods were predominantly Hispanic (*N* = 46%) and White (*N* = 34%).

**Table 1:** Characteristics of pregnant patients stratified by neighborhood median household income

	Lower (*N* = 103) ^*^ N (%) or years (SD)	Upper (*N* = 103) * N (%) or years (SD)	*p*value
Age at delivery, years (mean) (standard deviation)	30.5 (6.3)	33.7 (5.1)	** < 0.001**
Maternal race/ethnicity			** < 0.001**
White (non-Hispanic)	35 (34)	49 (48)	
Asian	14 (14)	32 (31)	
Black (non-Hispanic)	1 (1)	0	
American Indian/Alaska Native/Native Hawaiian/Pacific Islander	2 (2)	2 (2)	
Hispanic	46 (45)	12 (12)	
Other	5 (5)	8 (8)	
Insurance			** < 0.001**
Public	47 (46)	20 (19)	
Private	56 (54)	83 (81)	
Gravida			0.16
1	42 (41)	53 (51)	
2–3	43 (42)	40 (39)	
4 +	18 (17)	10 (10)	
Parity			**0.022**
0	55 (53)	69 (67)	
1–2	38 (37)	32 (31)	
3 +	10 (10)	2 (2)	
Cardiac diagnoses ^†^			
Structural^‡^	36 (35)	20 (19)	0.02
Cardiomyopathy	18 (17)	17 (17)	> 0.99
Congenital	40 (39)	32 (31)	0.31
Arrhythmia	31 (30)	46 (45)	0.04
Other	24 (23)	16 (16)	0.21
Limited prenatal care	4 (4)	1 (1)	0.40
Late initiation of prenatal care	3 (3)	2 (2)	1
Cardiologist established prior to pregnancy			0.89
Yes	85 (83)	83 (81)	
Unknown	1 (1)	2 (2)	
Chronic condition prior to pregnancy (other than cardiac disease) ^†^			
Any chronic condition	78 (76)	73 (71)	0.53
Hypertension	11 (11)	14 (14)	0.67
Pulmonary disease	15 (15)	10 (10)	0.39
Neurological	17 (17)	18 (17)	1.0
Autoimmune disorder	9 (9)	12 (12)	0.65
Diabetes (pregestational)	8 (8)	1 (1)	0.04
Prior thrombotic disorder	3 (3)	4 (4)	0.99
Anxiety/depression	15 (15)	20 (19)	0.46
Chronic pain	6 (6)	3 (3)	0.50
Renal disease	0	2 (2)	0.50
Gastrointestinal	6 (6)	16 (16)	0.04
Hyperlipidemia	7 (7)	4 (4)	0.54
Obesity	9 (9)	8 (8)	1
Genitourinary	0	6 (6)	0.03
Malignancy	0	1 (1)	0.99
Other	41 (40)	36 (35)	0.56

Bold font remains for significant *p* values < 0.05

^*^Lower income groups were defined as median household incomes ranging from $40,452 to $117,264. Upper income group was defined as $117,265 to $250,000 +

^†^Some patients had multiple diagnoses

^‡^Structural diagnoses included diagnoses like aneurysm, valvular stenosis/prolapse/insufficiency, mechanical valve, aortic valve/root dilation, and myxomatous valve

^§^Defined as less than 3 obstetric clinic prenatal visits

^||^Defined as prenatal care initiated after 24 weeks gestation

*Abbreviation*: *IQR*, interquartile range; *SD*, standard deviation

Individuals living in lower income neighborhoods were more likely to have public insurance (46% vs 19%, *p* < 0.001). There was no significant difference in the prevalence of any non-cardiac chronic condition between neighborhoods (76% in the lower income group and 71% in the upper income group). However, the distribution of types of chronic conditions was significantly different between the two groups (*p* = 0.03) with patients in low-income neighborhoods having more pregestational diabetes (*N* = 8% vs 1%, *p* = 0.04) and less gastrointestinal (*N* = 6% vs 16%, *p* = 0.04) or genitourinary (*N* = 0% vs 6%, *p* = 0.03) conditions, although the rates of any specific disease were low in both groups.

Table 2 describes the primary outcomes of intrapartum and postpartum cardiac events and preterm delivery. There was no significant difference in the rates of intrapartum cardiac events (10% in lower income vs. 4% in upper income, *p* = 0.16) or postpartum cardiac events (14% in lower income vs. 17% in upper income, *p* = 0.70). There was also no difference in preterm delivery rates (24% in lower income vs. 17% in upper income, *p* = 0.23). However, patients living in lower income neighborhoods did have higher rates of antepartum hospital admissions (42% vs. 22%, *p* = 0.004).

**Table 2:** Outcomes of pregnant patients stratified by neighborhood median household income

Outcomes	Lower (*N* = 103) ^*^ N (%)	Upper (*N* = 103) ^*^ N (%)	*p* value
Primary outcomes			
Intrapartum cardiac/thrombotic event	10 (10)	4 (4)	0.16
Postpartum cardiac/thrombotic events	14 (14)	17 (17)	0.70
Preterm delivery	25 (24)	17 (17)	0.23
Secondary outcomes			
Preeclampsia	15 (15)	7 (7)	0.11
Gestational diabetes	16 (16)	13 (13)	0.82
Preterm labor	17 (17)	10 (10)	0.21
Postpartum breastfeeding	94 (91)	96 (93)	0.80
Postpartum hemorrhage	8 (8)	4 (4)	0.37
Type of delivery			0.92
Cesarean	37 (36)	40 (39)	
Assisted (forceps, vacuum)	13 (13)	13 (13)	
NSVD	53 (51)	50 (49)	
Antepartum hospitalization	43 (42)	23 (22)	**0.004**
Antepartum hospitalization reasons ^†^			
Obstetric	18 (17)	15 (15)	0.28
Cardiac	24 (23)	16 (16)	0.83
Other ^‡^	18 (17)	6 (6)	0.34
Unknown	1 (1)	0	0.99
Postpartum cardiac/thrombotic events	14 (14)	17 (17)	0.70
Prolonged hospitalization postpartum	21 (20)	15 (15)	0.36
NICU admission after delivery	21 (20)	16 (16)	0.47
Small for gestational age	15 (15)	9 (9)	0.28
Maternal death	0	0	–
Neonatal death	4 (4)	1 (1)	0.37

Bold font remains for significant *p* values < 0.05

^a^Lower income groups were defined as median household incomes ranging from $40,452 to $117,264. Upper income group was defined as $117,265 to $250,000 +

^†^Some patients had multiple hospitalizations for different reasons

^‡^Other reasons for antepartum hospitalization included infections, falls, mental health (substance abuse, suicidality), and vertigo

*Abbreviation*: *NICU*, neonatal intensive care unit; *NSVD*, normal spontaneous vaginal delivery

There were 0 maternal deaths, 5 neonatal deaths, and 0 intrauterine fetal demise. Two of the neonatal deaths were due to congenital anomalies, 1 from extreme premature birth in the setting of cervical insufficiency, 1 from sepsis, and 1 after placental abruption.

## Discussion

We evaluated the impact of neighborhood-based income on cardiac and obstetric outcomes in pregnant women with pre-existing heart disease who were being cared for in a multidisciplinary cardio-obstetrics program. The salient findings of our study are (1) there are significant demographic differences between patients living in different neighborhoods, with those in lower income neighborhoods being younger, more likely to be Hispanic, and having more pregestational diabetes. (2) Antepartum hospitalization is significantly different between lower and upper income neighborhoods, with those in lower income neighborhoods being more likely to require hospitalization prior to childbirth. (3) Neighborhood-based income does not significantly impact cardiac or obstetric outcomes when patients are treated at a multidisciplinary cardio-obstetrics program. This suggests that these programs may be able to play some role in mitigating baseline, previously reported socioeconomic differences by providing early access to quaternary level care and closer monitoring.

Overall, our results did not show a significant difference in the primary outcome. The high rate of antepartum hospitalization for patients in lower income neighborhoods may represent the identification of higher risk patients who are hospitalized before decompensation. The cardio-obstetrics multidisciplinary program (including high-risk obstetrics, anesthesia, cardiology, cardiovascular surgery, social work, and nursing) at our institution meets monthly to review all patients. Programs like this, which are focused specifically on care for the cardiac-obstetrics patient, are well positioned to identify high-risk patients and may improve chances of intervening at an early stage to prevent adverse outcomes [12, 35]. Another potential cause of differences in antepartum hospitalizations includes distance from the hospital, and higher admission rates may reflect a concern about lack of follow-up or an inability to be clinically reassessed. Other studies have correlated the neighborhood one lives in with pregnancy outcomes. For example, “physical disorder” (e.g., trash, abandoned vehicles) in a pregnant patient’s neighborhood has been associated with greater adverse pregnancy outcomes [36].

Our research confirms and extends the limited published data of cardio-obstetric programs. A similar study was recently published in 2020 on 306 pregnant individuals in a quaternary care hospital in New York City [20]. That study’s population was predominantly White, Black, and Hispanic, with no Asian patients recorded, while our database is significant for a high percentage of Asian individuals. Their population had 74.2% of patients on Medicaid versus our study, which had an overall rate of public insurance of 32.5%. Our overall rates of cardiac antepartum hospitalization (*N* = 40, 19%) were not significantly different from theirs, which had 48 (15.7%) cardiovascular-related antepartum admissions. This study did not specifically compare outcomes but provided an overall description of the patient population.

Our overall rates of cardiac events were 7% (intrapartum) and 15% (postpartum), and there was no significant difference between patients in the upper and lower income neighborhoods. This can be compared with a population-based study of mothers with heart disease delivering in New York City that found a major adverse cardiac event rate of 16.1% [5]. Our overall rate of preterm delivery was 20%, which is higher than the average preterm birth rate of 9% in California [37]. A large, international retrospective registry of pregnancies in individuals with heart disease reports preterm birth rates of 15.8% (*n* = 905) [38].

There are several strengths of our work. Our study includes the full population of cardio-obstetric patients seen at this institution and not a subset. Additionally, our data were obtained through chart review instead of questionnaires, which avoids missing data in responses. Finally, our data add to the limited literature on cardio-obstetric patients. In particular, we have contributed data with a higher percent of Asian individuals, while other data are primarily focused on Black and White patients.

There are also several limitations of our work. The retrospective study design prevented assessment of outcomes occurring after discharge from the delivery hospitalization. We used all available patients in the retrospective cohort, but the sample size was limited, particularly for assessing differences in rare outcomes. Further, it is possible that some patients in our sample had more severe presentation of diseases, which may have resulted in differences in outcomes (although the overall incidence of chronic conditions before pregnancy is comparable between groups). We utilized zip code residence information for our patients and did not have direct income information. While there is precedent for using median household income per zip code as a relative comparison of the resident’s socioeconomic status [39–42], it is less granular than patient-specific data or addresses. The annual median household income of the neighborhoods included in our analysis was $117,264. While this is significantly above national poverty levels, the cost of living in this area is high. The California family needs calculator estimates that the poverty level for a family (consisting of two adults and two school-aged children) in the county where our hospital is located is $93,737 [43]. However, we recognize that wealth density and resources available in this area are not representative of all patients. Finally, this was based on the experience of a single quaternary care center in Northern California. These results may not be applicable to other states or regions.

## Conclusion

In pregnant individuals with pre-existing heart disease, neighborhood-based income is associated with younger age, minority status, and antepartum hospitalization. However, we found no difference in cardiac or obstetric outcomes, possibly reflecting the influence of a cardio-obstetrics program in reducing outcome disparities that have previously been reported. Further research with larger, more diverse sample sizes is recommended to elucidate the role such programs play in improving health outcomes and eliminating health disparities.

## Data Availability

Anonymized data may be made available at request.

## Code Availability

NA.
